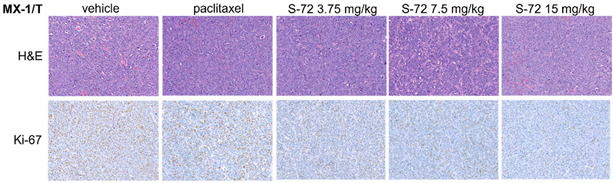# Correction: Hou et al. S-72, a Novel Orally Available Tubulin Inhibitor, Overcomes Paclitaxel Resistance via Inactivation of the STING Pathway in Breast Cancer. *Pharmaceuticals* 2023, *16*, 749

**DOI:** 10.3390/ph18091355

**Published:** 2025-09-10

**Authors:** Zhenyan Hou, Songwen Lin, Tingting Du, Mingjin Wang, Weida Wang, Shen You, Nina Xue, Yichen Liu, Ming Ji, Heng Xu, Xiaoguang Chen

**Affiliations:** 1 State Key Laboratory of Bioactive Substance and Function of Natural Medicines, Institute of Materia Medica, Chinese Academy of Medical Sciences and Peking Union Medical College, Beijing 100050, China; houzhenyan@imm.ac.cn (Z.H.); linsongwen@imm.ac.cn (S.L.); ninadu@imm.ac.cn (T.D.); wangmingjin@imm.ac.cn (M.W.); wangweida@imm.ac.cn (W.W.); youshen@imm.ac.cn (S.Y.); angelnina@imm.ac.cn (N.X.); liuyichen@joinn-lab.com (Y.L.); 2 Beijing Key Laboratory of New Drug Mechanisms and Pharmacological Evaluation Study, Institute of Materia Medica, Chinese Academy of Medical Sciences and Peking Union Medical College, Beijing 100050, China; 3 Beijing Key Laboratory of Non-Clinical Drug Metabolism and PK/PD Study, Institute of Materia Medica, Chinese Academy of Medical Sciences and Peking Union Medical College, Beijing 100050, China

## Error in Funding

In the original publication [1], there are errors in the Funding Number. The authors state that the scientific conclusions are unaffected. The corrected version appears below. This correction was approved by the Academic Editor. The original publication has also been updated.

**Funding:** This work was supported by the National Natural Science Foundation of China (grant nos. 82104202 and 81903904) and the CAMS Innovation Fund for Medical Science (CIFMS) (CAMS Innovation Fund for Medical Science (CIFMS) (2021-I2M-1-026)).

## Error in Figure

In the original publication [1], there was a mistake in Figure 4F as published. The HE data in the paclitaxel group was missing and unintentionally pasted as that of the vehicle group due to carelessness. The corrected Figure 4F appears below. The authors state that the scientific conclusions are unaffected. This correction was approved by the Academic Editor. The original publication has also been updated.