# Supplementary material for: S-72, a Novel Orally Available Tubulin Inhibitor, Overcomes Paclitaxel Resistance via Inactivation of the STING Pathway in Breast Cancer
Source: Pharmaceuticals (Basel). 2023 May 15;16(5):749. doi: 10.3390/ph16050749 (PMC10221130; doi:10.3390/ph16050749)
Supplement: Supplementary file 1 [file pharmaceuticals-16-00749-s001.zip › pharmaceuticals-2312893-supplementary.pdf]

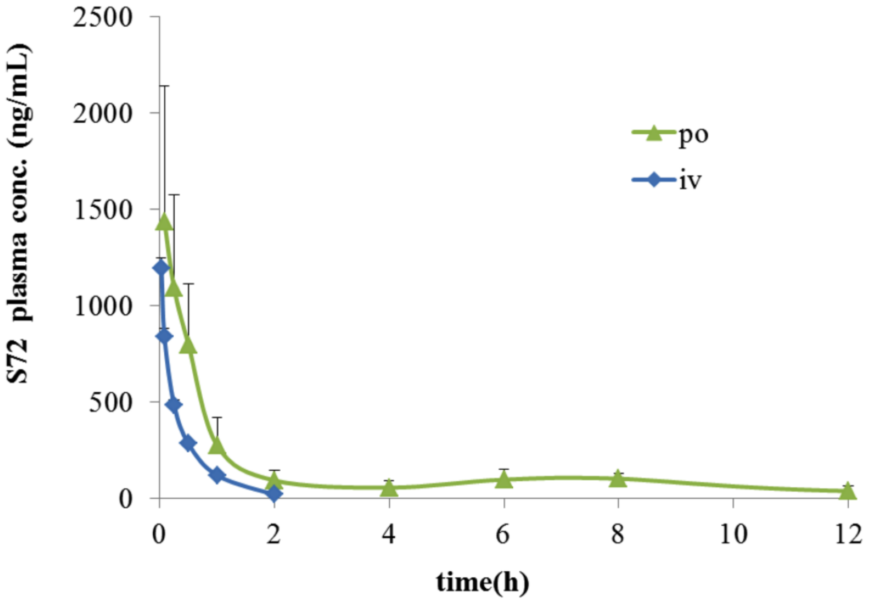

Figure S1. Plasma concentration-time curves of S-72. ICR mice were orally administered 5 mg/kg S-72 or intravenous injected 1 mg/kg S-72. Plasma concentrations of S-72 were analyzed by a LC-MS/MS method.

A

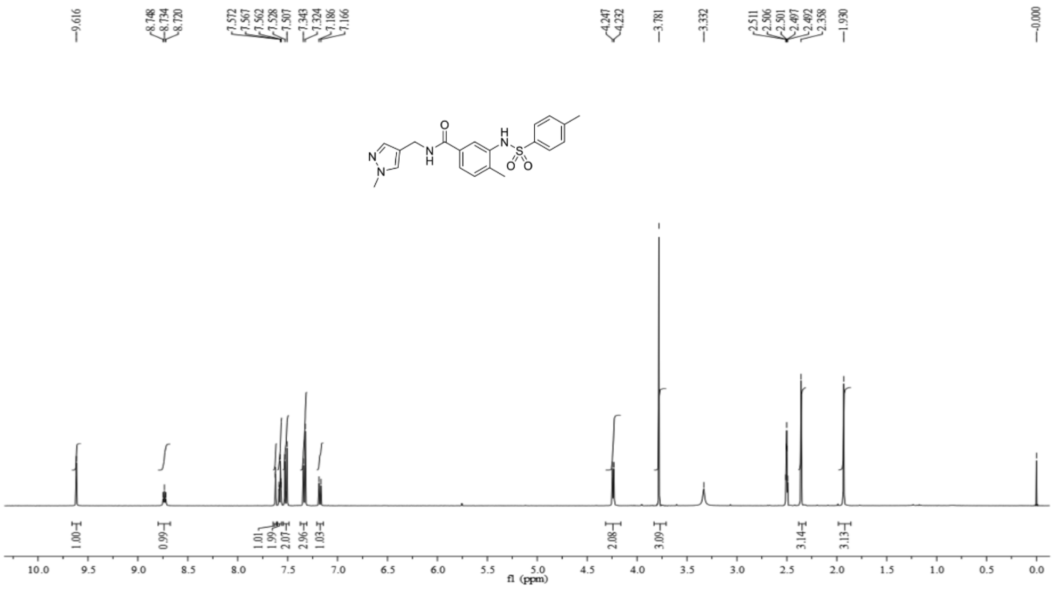

B

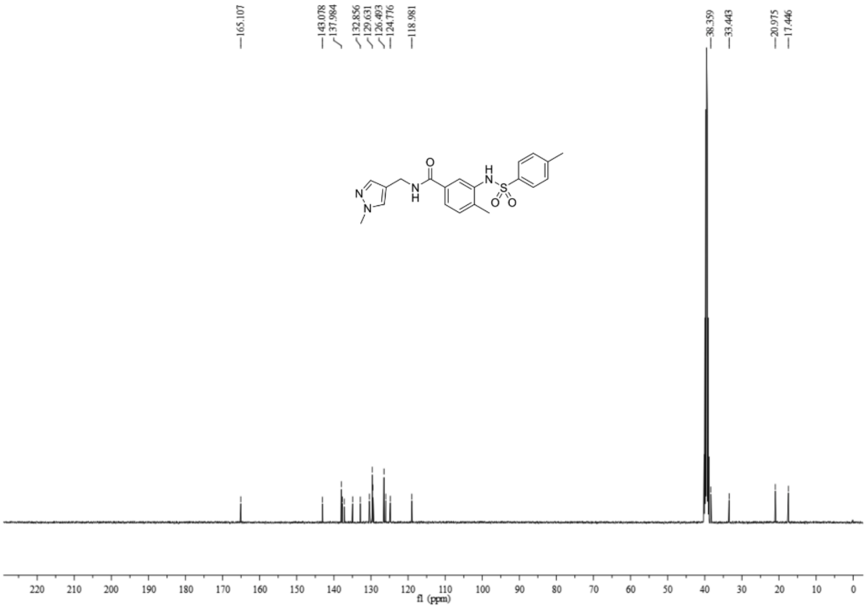

Figure S2. (A) HNMR and (B) CNMR spectrum of S-72.

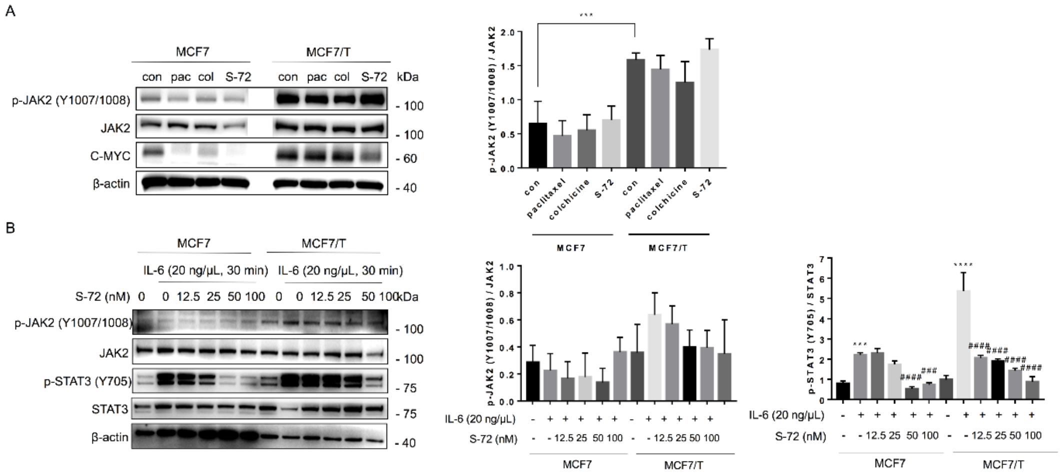

Figure S3. S-72 inhibits STAT3 activation in both MCF7 and MCF7/T cells. (A) Protein levels of p-JAK2 (Y1007/1008), JAK2 and C-MYC were identified by western blot after treatment with 100 nM paclitaxel, 100 nM colchicine or 100 nM S-72 for 24 h. (B) S-72 concentration-dependently inhibited STAT3 phosphorylation at Tyr705 induced by IL-6 in MCF7 and MCF7/T cells. *N* = 3, $^{*}p < 0.05$, $^{**}p < 0.01$, $^{***}p < 0.001$, $^{****}p < 0.0001$ versus con and $^{\#}p < 0.05$, $^{\#\#}p < 0.01$, $^{\#\#\#}p < 0.001$, $^{\#\#\#\#}p < 0.0001$ versus the IL-6 group.

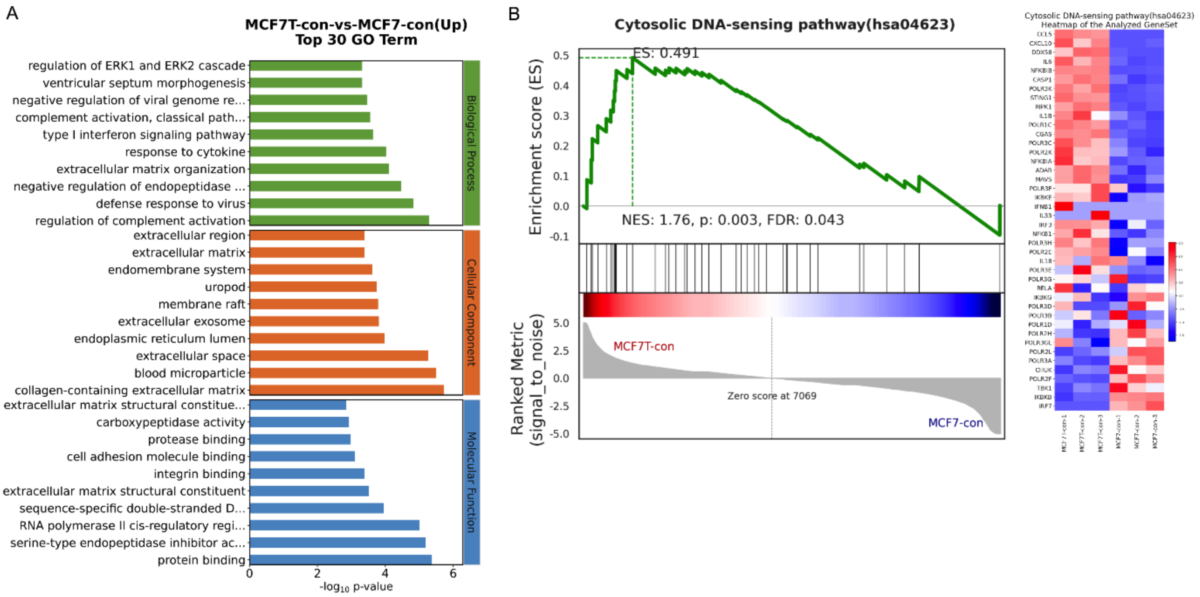

Figure S4. RNA-Seq analysis shows that paclitaxel-resistant MCF7/T cells have overexpressed cGAS/STING signaling pathways compared with MCF7 cells. (A) GO analysis performed on differentially expressed genes in MCF7/T cells compared with MCF7 cells. (B) GSEA analysis on KEGG enriched gene set.

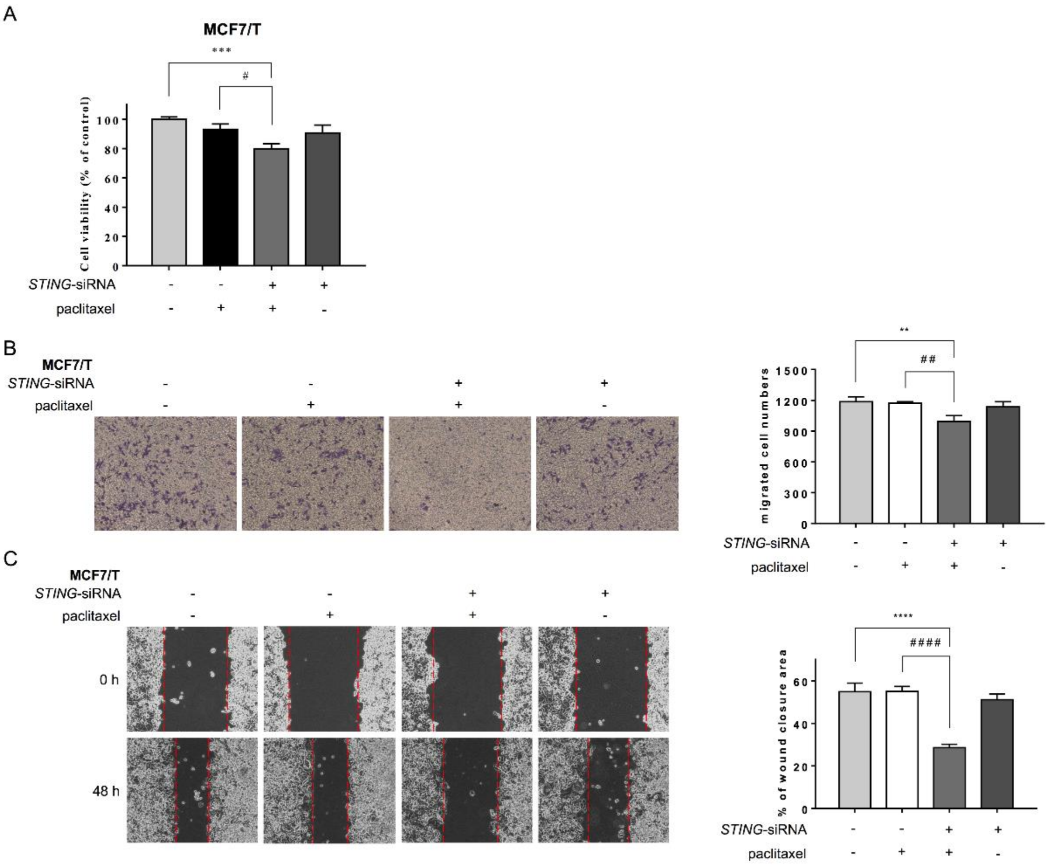

Figure S5. Knockdown of STING restores sensitivity to paclitaxel in MCF7/T cells. (A) Cell viability determined by CCK8 assay. (B-C) Effects of STING knockdown on paclitaxel sensitivity evaluated by (B) transwell migration assay and (C) wound healing assay. $N = 3$, $^{*}p < 0.05$, $^{**}p < 0.01$, $^{***}p < 0.001$, $^{****}p < 0.0001$ versus the control group and $^{\#}p < 0.05$, $^{\#\#}p < 0.01$, $^{\#\#\#}p < 0.001$, $^{\#\#\#\#}p < 0.0001$ versus the paclitaxel-treated group.

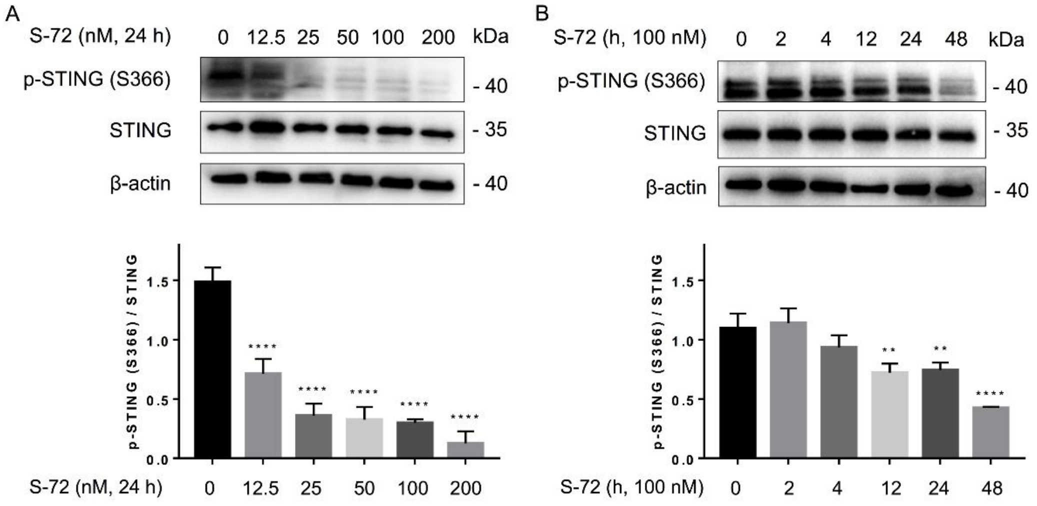

Figure S6. Changes in protein levels of p-STING (Ser366) after S-72 treatment. (A) Dose course study on the effect of S-72 treatment on p-STING (Ser366) expressions in MCF7/T cells. Cells were treated with gradient concentrations of S-72 for 24 h. (B) Time course study on the effect of S-72 treatment on p-STING (Ser366) expressions in MCF7/T cells. Cells were treated with 100 nM S-72 for various time periods. Protein levels were detected by western blot analysis. $N = 3$, $^{*}p < 0.05$, $^{**}p < 0.01$, $^{***}p < 0.001$, $^{****}p < 0.0001$ versus con.

Table S1. Pharmacokinetic parameters of S-72.

Parameters	Units	po. (5 mg/kg)	iv. (1 mg/kg)
$t_{1/2\beta}$	h	4.07	0.38
T_{max}	h	0.083	0.03
C_{max}	ng/ml	1436	1192
$AUC_{(0-t)}$	h*ng/ml	1740	469
$AUC_{(0-\infty)}$	h*ng/ml	1962	480
$MRT_{(0-t)}$	h	3.26	0.42
$MRT_{(0-\infty)}$	h	4.92	0.46
Vz	ml/kg	-	1155
CL	ml/h/kg	-	2084
F%	-	74.1	-
